# Development of an atmospheric plasma jet device for versatile treatment of electron microscope sample grids

**DOI:** 10.1016/j.jbc.2022.101793

**Published:** 2022-03-04

**Authors:** Eungjin Ahn, Tianyu Tang, Byungchul Kim, Hae June Lee, Uhn-Soo Cho

**Affiliations:** 1 Department of Biological Chemistry, University of Michigan, Ann Arbor, Michigan, USA; 2 Department of Electrical Engineering, Pusan National University, Busan, South Korea

**Keywords:** low-temperature plasma, grid treatment, Raman spectroscopy, single particle analysis, negative-stain electron microscopy, cryo-electron microscopy, AC, alternating current, AFM, atomic force microscopy, cryo-EM, cryogenic electron microscopy, DC, direct current, LTP, low-temperature plasma, *M. caps* sMMOH, *Methylococcus capsulatus* soluble methane monooxygenase, Neg-EM, negative-stain electron microscopy, SLM, standard liter per min

## Abstract

Atmospheric-pressure plasmas have been widely applied for surface modification and biomedical treatment because of their ability to generate highly reactive radicals and charged particles. In negative-stain electron microscopy (Neg-EM) and cryogenic electron microscopy (cryo-EM), plasmas have been used to generate hydrophilic surfaces and eliminate surface contaminants to embed specimens onto grids. In addition, plasma treatment is a prerequisite for negative-stain and Quantifoil grids, whose surfaces are coated with hydrophobic amorphous carbon. Although the conventional glow discharge system has been used successfully in this purpose, there has been no further effort to take an advantage from the recent progress in the plasma field. Here, we developed a nonthermal atmospheric plasma jet system as an alternative tool for treatment of surfaces. The low-temperature plasma is a nonequilibrium system that has been widely used in biomedical area. Unlike conventional glow discharge systems, the plasma jet system successfully cleans and introduces hydrophilicity on the grid surface in the ambient environment without a vacuum. Therefore, we anticipate that the plasma jet system will have numerous benefits, such as convenience and versatility, as well as having potential applications in surface modification for both negative-stain and cryo-EM grid treatment.

Plasma treatment for materials processing has a long history and has been successfully used in the semiconductor industry. For the last couple of decades, low-temperature plasmas (LTPs) under atmospheric pressure have been applied to a wide range of biomedical and surface-treatment applications because they can control the chemical reactions of radicals and change the energy distributions of charged particles (1, 2, 3). LTPs have nonequilibrium electrons and ions, where the gas and ion temperatures are relatively lower than those of a conventional thermal plasma to avoid thermal damage to the specimen, while the electron temperature is up to tens of thousands kelvin (4). LTP is a versatile tool that can control and introduce hydrophilicity into a carbon surface by minor oxidation and surface cleaning without causing severe damage to the original chemical structures. This technique is already widely used for cleaning and hydrophilic modification during protein sample preparation (*i.e.*, glow discharge) in the biochemical research field (5).

Determining atomic-resolution protein structures using single-particle cryogenic electron microscopy (cryo-EM) has become increasingly routine and vital in the field of structural biology (6). Recent technical advances in cryo-EM instruments (cold field-emission gun, aberration corrector, next-generation direct electron detector, etc.) and software developments have further accelerated this trend (7, 8, 9, 10). Therefore, it is not surprising that sample and grid preparation has become a bottleneck in elucidating the protein structure using cryo-EM. Purified biomolecular assemblies are embedded in thin amorphous ice after plunge-freezing in liquid ethane. Successful microscopic data acquisition requires the optimization of specimen preparation procedures, such as selecting grid types/treatment and finding the best blotting conditions (11, 12). Various types of grids are commercially available, which have been developed to overcome ice thickness, beam-induced particle motion during image collection (13), and protein denaturation due to exposure at the air–water interface (14, 15, 16). Regardless of the grid type, plasma treatment is a prerequisite step before applying a protein specimen (17). Because the grid surface is hydrophobic and contaminated by dirt and adsorbents (18), plasma treatment is necessary to clean and modify the grid surface into a hydrophilic surface, thereby enhancing grid and solution contact. In particular, negative-stain electron microscopy (Neg-EM) grids and Quantifoil grids are additionally coated with hydrophobic carbons, which must be modified to form a hydrophilic surface before the specimen can be applied (19).

The low-energy plasma modification system is a widely used instrument for grid pretreatment to introduce hydrophilicity and to clean the grid surface under vacuum. Recently, the plasma treatment step has been combined with vapors of chemical precursors to further introduce functional groups on the graphene surface to overcome the preferred orientation and reduce specimen movement (20). Although this new approach appears promising for functionalizing the graphene surface in a controlled manner, it is very challenging to build such a device as an individual laboratory. Here, we introduce an atmospheric-pressure plasma jet device that generates plasma in an air environment with the potential for versatile surface modification. The strengths of the plasma jet system are as follows: First, it is easy to install at a low price. Second, it is easy to use because it does not require a vacuum. Finally, with the assistance of additional systems, it has the potential for surface modification with simple chemical molecules. We anticipate that this plasma jet device will greatly contribute to the development of functional grids and single-particle protein observations in cryo-EM.

## Results and discussion

### Building the atmospheric-pressure plasma jet device

Plasma is described as a quasineutral mixture of charged particles and radicals in a partially ionized gas. These activated gas molecules possess high reactivity toward hydrocarbon surfaces, oxidizing and making them hydrophilic for further applications. In general, surface treatment by plasma requires a vacuum environment to increase the carry distance (*i.e.*, mean free path) of activated ionic gas molecules; otherwise, they can be eliminated before arriving at the target surface because of their high collision rate and reactivity with other gas molecules. The mean free path of an ionized gas molecule can be calculated using Equation 1 (Equation 1), where ${\text{λ}}_{i}$ is the mean free path of gas molecule i, k is the Boltzmann constant (1.38 × 10^−23^ J/K), T and p are the temperature and pressure of the environment, respectively, and ${\text{d}}_{i}$ is the diameter of gas molecule i.

(1) $${\text{λ}}_{i}=\frac{k*T}{\sqrt{2}*\text{π∗}{\text{d}}_{i}²\text{∗p}}$$

**Figure 1:**
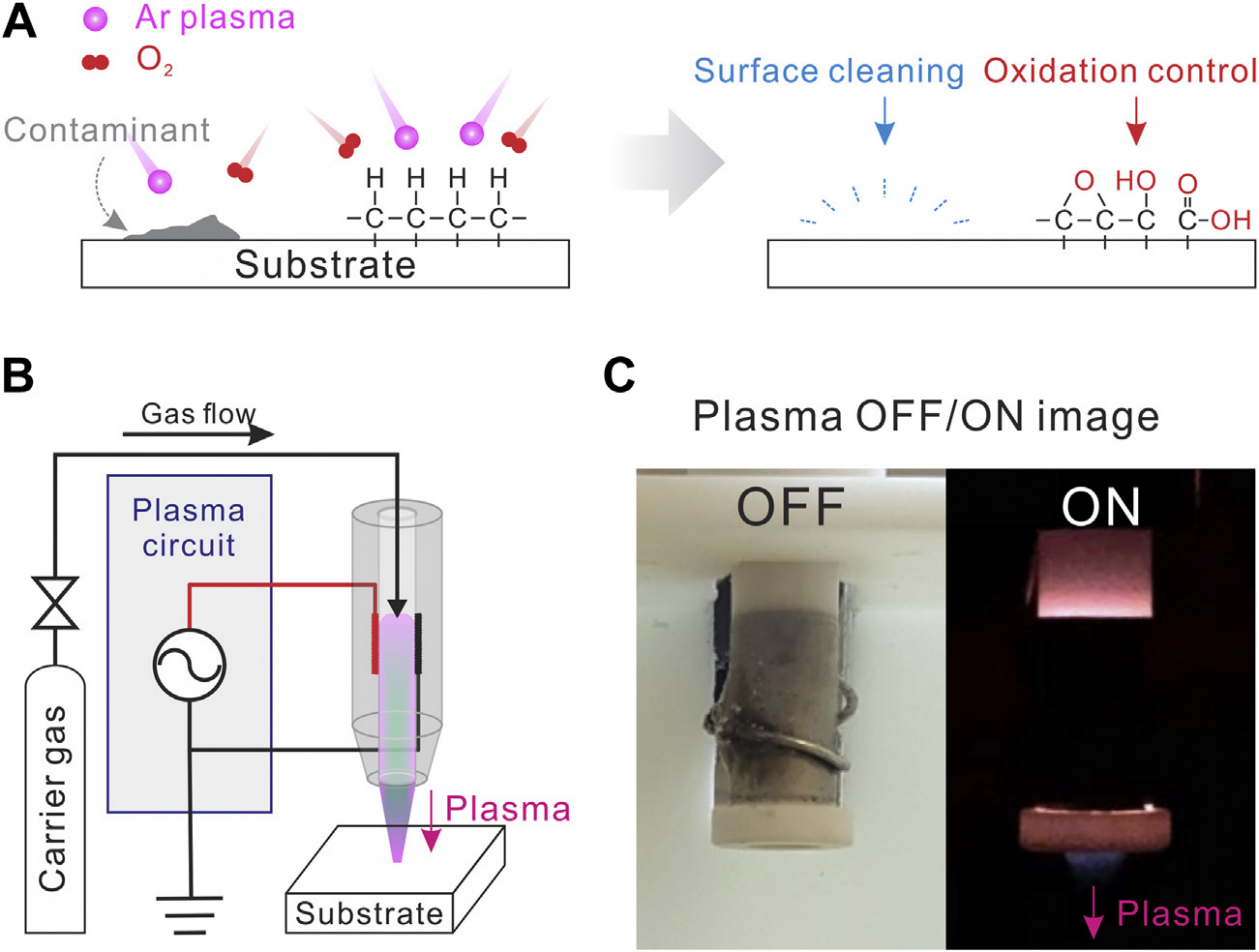
**Overview of the plasma jet system.***A*, schematic of the effect of plasma treatment on a hydrocarbon surface. *B*, schematic of the overall plasma jet system identifying each component. Photographs of the overall system and each component are provided in the Fig. S1. *C*, photographs of the dielectric jet (*left*) and plasma ejected from the jet under a dark background (*right*).

For example, the mean free path of activated argon (Ar) molecules under ambient conditions (298 K, 1 atm) is shorter than 70 nm, while it can be increased up to 300 μm in a vacuum environment (298 K, 0.20 mbar). The plasma jet system helps charged particles reach the target surface by pumping with a carrier gas before undergoing unfavorable reactions in the atmosphere, thereby achieving surface modification even in an atmospheric environment. Building the plasma jet system only requires a few parts, such as a DC voltage source (*i.e.*, power source), high-voltage DC–AC inverter circuit (*i.e.*, plasma circuit), dielectric tube (*i.e.*, plasma jet), and gas flow controlled with a regulator (Figs. 1*A* and S1). Therefore, the plasma jet system does not require a vacuum pump or chamber and works well in an atmospheric environment, which reduces the operation time by eliminating the warm-up period. We believe that our plasma jet system is cheaper and easy to set up without professional knowledge or experience.

Typical chemical reactions in the plasma jet tube are as follows. First, the Ar carrier gas is activated into either cationic (Ar^+^) or radical (Ar∗) forms *via* the high voltage generated by the AC circuit through chemical reactions R1 and R2 (excited species are shown in bold for clarity) (21):

(R1) $$Ar+e^{-}\overset{\;}{\to }\;Ar^{+}+2e^{-}$$

(R2) $$Ar+e^{-}\overset{\;}{\to }\;Ar^{*}+e^{-}$$

Then, the activated Ar species with an energy larger than the ionization threshold of the O_2_ molecule generate activated oxygen species (R3 and R4) (22, 23), while the excess energy is released in the form of light (R5):

(R3) $$Ar^{*}+O_{2}\overset{\;}{\to }\;O+O^{*}+Ar$$

(R4) $$Ar^{+}+O_{2}\overset{\;}{\to }\;O_{2}^{+}+Ar$$

(R5) $$Ar^{*}\overset{\;}{\to }\;Ar+hv$$

When the excited oxygen species reach the hydrocarbon surface contaminated by dirt and adsorbents, gradual oxidation occurs to remove these contaminants (18). It also generates functional groups (*e.g.*, hydroxyl, epoxy, and carboxyl groups), which convert the hydrocarbon surface into a polar and hydrophilic surface (Fig. 1*A*). The plasma jet is activated when a minimum power (0.55 W) is applied to the AC circuit with a gas flow injected from the tube to the surface (Fig. 1, *B* and *C*). The detailed interior structure of the jet and the applied voltage *versus* discharge current waveforms of the Ar plasma jet are shown in Fig. S2. The most convenient method to find the power consumed by the plasma jet device is to integrate the Lissajous curve using the current/voltage characteristics and multiply this value by the operation time. The discharge power consumed by the Ar plasma jet with an input power of 1.07 W was calculated to be 0.43 W by the Lissajous figure method with a 0.1 μF external capacitor.

### Hydrophilicity and surface cleaning induced by the plasma jet

**Figure 2:**
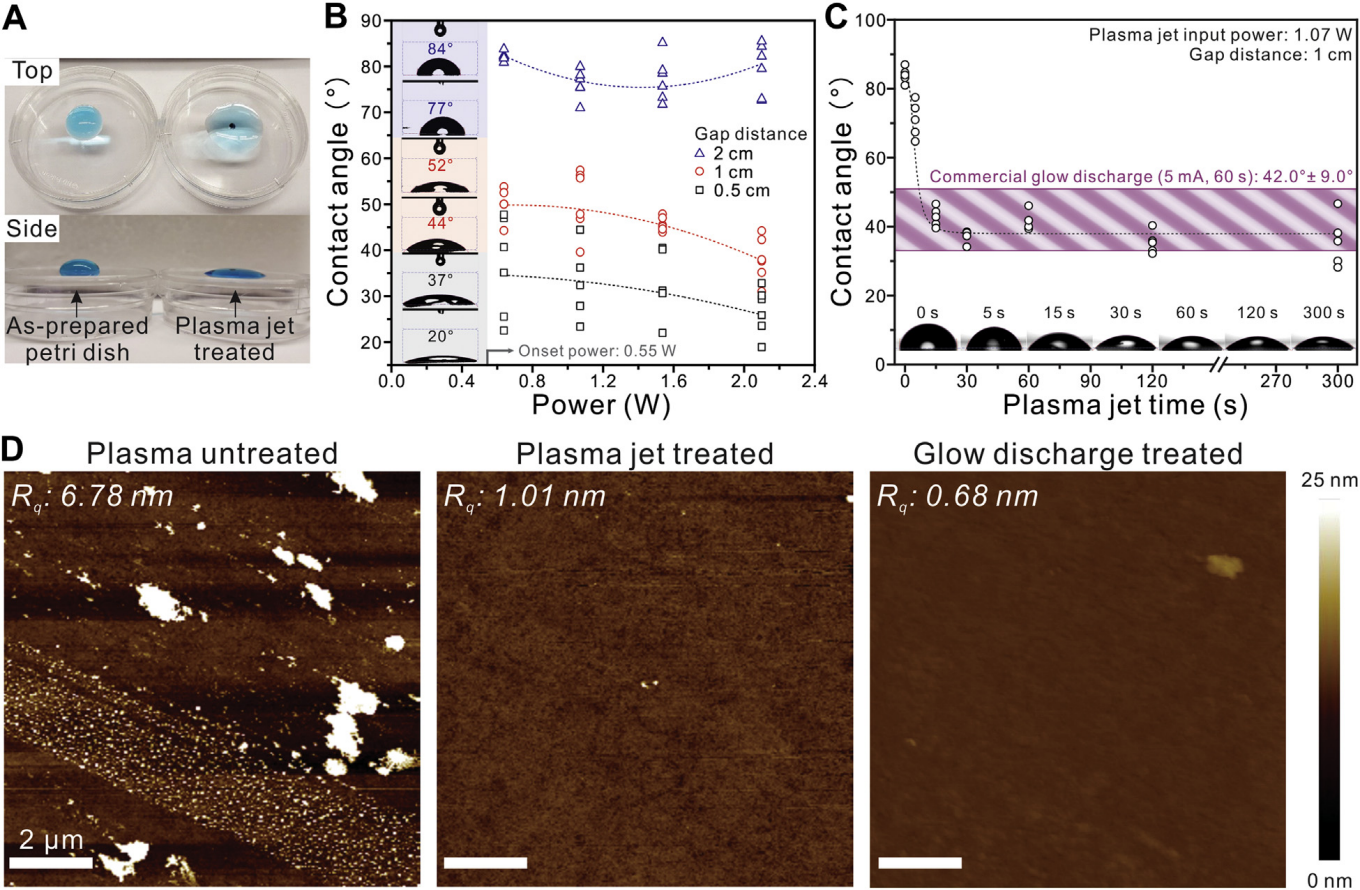
**Induced hydrophilicity and surface cleaning effect of the plasma jet system.***A*, change in the hydrophilicity of a Petri dish before and after plasma jet treatment (2 min). The water droplet was colored with methylene *blue* to aid visualization. *B* and *C*, water contact angle measured by a contact angle goniometer on the Petri dish surface after plasma jet treatments (*B*) with different input powers and gap distances and (*C*) over time with a fixed input power (1.07 W) and gap distance (1 cm). *Dotted line* denotes the fitted curve of averages. The *violet* box denotes the average contact angle of the Petri dish treated by a commercial glow discharge system (5 mA, 60 s). Each contact angle is the average of five measurements in different locations. Insets: Photographs of water droplets with the measured contact angle and plasma jet treatment time. *D*, comparison of the AFM morphology of slide glass before (plasma untreated) and after plasma jet and glow discharge treatments. AFM, atomic force microscopy.

The induced hydrophilicity and surface cleaning effect were compared between the plasma jet and a commercial glow discharge system (PELCO easiGlow, vacuum pressure <0.26 mbar). After plasma jet treatment of a Petri dish, the water droplet spread across the surface owing to the increased hydrophilicity (Fig. 2*A*). To monitor the degree of surface modification from plasma jet treatment, a water contact angle goniometer was employed. The gap distance between the jet and the specimen was altered along with the input power (Table S1 and Fig. S3). The hydrophilicity of the Petri dish increased with decreasing gap distance and increasing input power (Fig. 2*B*). A lower contact angle (*i.e.*, higher hydrophilicity) does not always enhance the biocompatibility of the surface with proteins (24). Therefore, a gap distance of 1 cm and a power of 1.07 W were chosen for the plasma jet treatment because this condition can induce a moderate level of hydrophilicity on the target surface, which is comparable with that induced by the commercial glow discharge system.

Time-dependent plasma jet treatment with a fixed gap distance (1 cm) and input power (1.07 W) showed that the contact angle of the plasma-jet-treated surface after 15 s of treatment was comparable to the measured contact angle of the glow-discharge-treated surface (5 mA, 60 s), which was 42° ± 9.0° (Fig. 2*C*) (25). Atomic force microscopy (AFM) was used to evaluate the surface cleaning effect after plasma jet treatment. The surface roughness and morphology of a glass slide were compared before and after plasma jet and glow discharge treatments (Fig. 2*D*). The surface roughness (R_q_) of the plasma untreated slide glass was 6.78 nm, which was reduced to 1.01 and 0.68 nm after plasma jet and glow discharge treatments, respectively. This indicates that the plasma jet system effectively cleans the surface and induces hydrophilicity, resulting in a surface that is comparable to that produced using a commercial glow discharge device without introducing a vacuum. When the plasma jet was applied to the negative-stain grid, the water droplet spread evenly across the surface of the grid, compared with the poor wetting exhibited before plasma treatment (Fig. S4). This implies that the specimen could be uniformly coated on the surface. This plasma-treated grid was subsequently used for protein observation using the negative-stain method.

### Oxidation studies of plasma treatment with graphene

Raman spectroscopy is a sensitive technique that detects the atomic vibrational modes in a chemical compound. In particular, the Raman spectroscopy is effective for understanding graphene, which shows sharp characteristic peaks based on atomic vibrations of the carbon lattice and defects. We adopted a graphene-based system to deeply understand the plasma jet device for two reasons. 1) The Raman behavior of graphene is sensitive to oxidation, which has been shown to increase the intensity of the defect peak. 2) Our ultimate use for the plasma jet was to further modify the hydrophilicity and degree of oxidation of graphene-coated EM grids.

**Figure 3:**
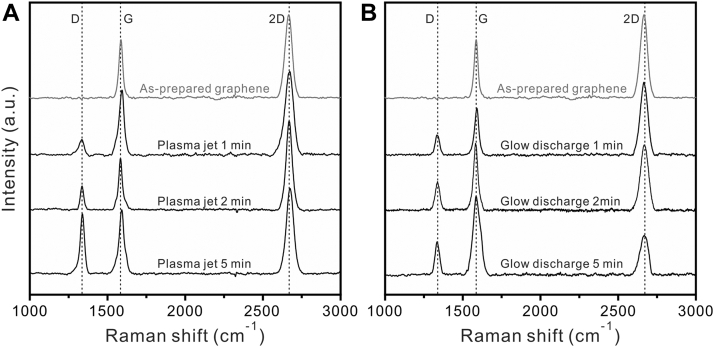
**Raman spectra of graphene monolayer after plasma jet and glow discharge treatment.** Raman spectra of graphene monolayers on Cu foil treated with the (*A*) plasma jet and (*B*) glow discharge systems. Three vertical *dotted lines* indicate the positions of the D, G, and 2D peaks from the graphene lattice.

To examine the oxidation behavior of the plasma jet system, we used commercially as-prepared monolayer graphene coated on a Cu foil (Graphenea) and monitored the variation in the degree and type of oxidation with the plasma treatment time. The plasma jet treatment exhibited an oxidation level comparable to that of the glow discharge system, with a gradually increasing D peak (1350 cm^−1^; defect-induced second-order Raman scattering) intensity with time, which indicates that the defect density increased upon exposure (Fig. 3, *A* and *B*). Both conditions similarly maintained the G peak (1580 cm^−1^; in-plane vibrations of sp^2^-hybridized carbon atoms), which indicates that the basal plane of graphene was maintained. The ratio between the intensities of the D and G peaks (D/G ratio), which indicates the relative oxidation degree compared with the crystalline carbon lattice in the graphene, increased from 0.24 to 0.75 with increasing plasma jet treatment time (from 1 to 5 min). This indicates that the plasma jet treatment provides a reliable and controllable oxidation effect. In addition, there was a notable difference in the 2D peak (2670 cm^−1^; a second-order overtone of a different in-plane vibration) between the plasma jet and glow discharge system for plasma treatment times over 2 min. After 5 min of glow discharge treatment, the graphene exhibited a less intense and broadened 2D peak, which indicates a reduction in the graphitic-ordered regions in the graphene lattice (26, 27) compared with that in the plasma-jet-treated graphene. Therefore, the Raman spectra analysis demonstrates that the plasma jet system modifies the surface of the graphene monolayer to introduce oxidation. Moreover, the plasma jet system better maintains the graphene lattice (2D peak) compared with the commercial glow discharge system. Therefore, the plasma jet introduces more oxidation with less damage to the graphene lattice compared with the commercial glow discharge system.

**Figure 4:**
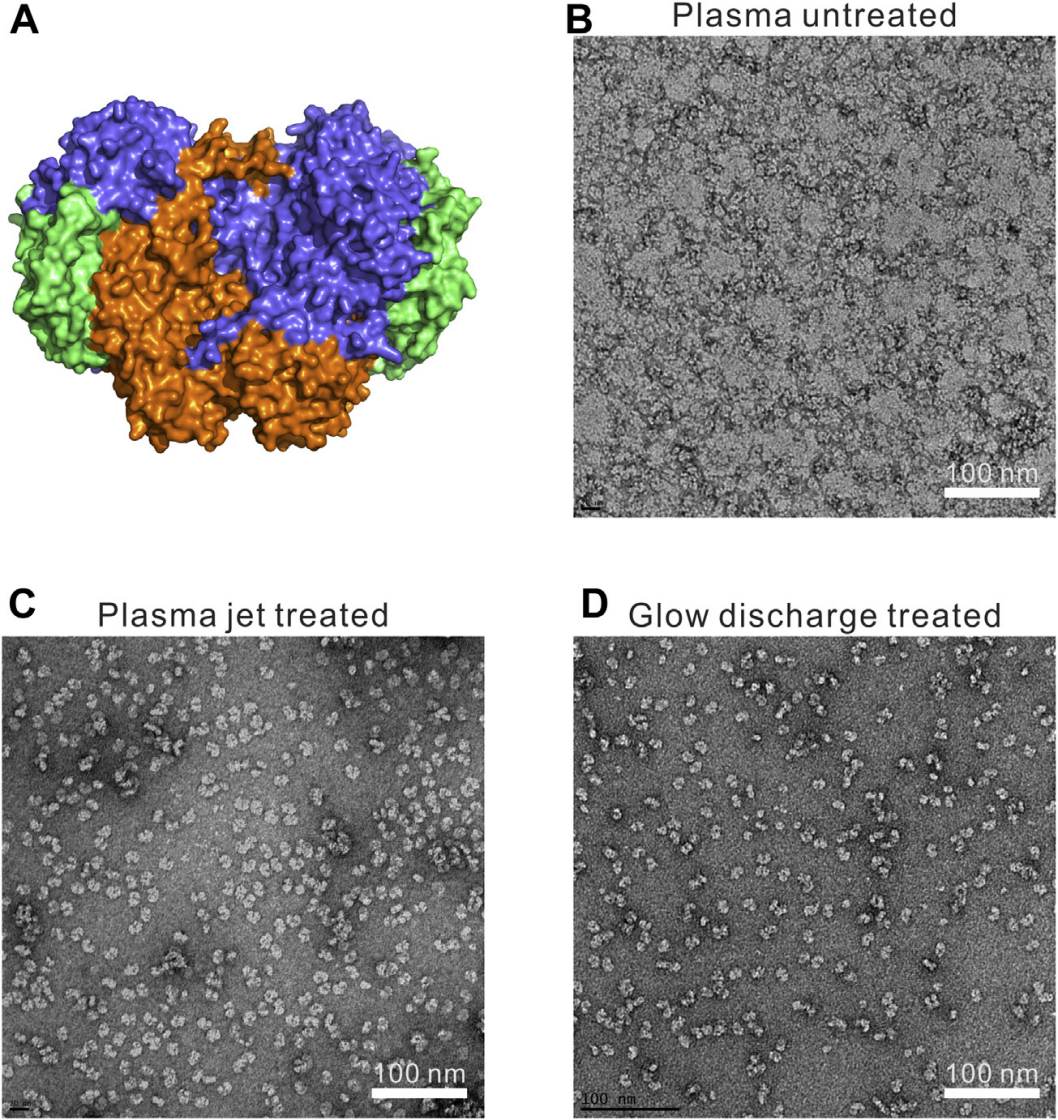
**The effect of plasma jet treatment on Neg-EM grids.***A*, surface representation image generated from the crystal structure of *M. caps* sMMOH (PDB ID: 1MTY) (32). Subunits of MMOHα, MMOHβ, and MMOHγ are colored *blue*, *orange*, and *green*, respectively. *B*–*D*, negative-stain EM images of *M. caps* sMMOH on (*B*) plasma untreated, (*C*) plasma-jet-treated, and (*D*) glow-discharge-treated negative-stain grids. M. caps sMMOH, Methylococcus capsulatus soluble methane monooxygenase.

### Comparison of Neg-EM images using the conventional glow discharge system and the plasma jet system

To study the practical application of the plasma jet system on EM grids, we employed the Neg-EM approach. Neg-EM grids were coated with amorphous carbon, which maintained the hydrophobic surface. The plasma treatment introduced hydrogenation on the carbon-coated EM grids, thereby allowing the specimen solution to make direct contact with the grid surface. Three different grids—plasma untreated, plasma jet treated (1.07 W, 60 s), and glow discharge treated (5 mA, 60 s)—were prepared, on which a protein sample (*Methylococcus capsulatus* soluble methane monooxygenase hydroxylase, *M.* *caps* sMMOH) was applied using the standard protocol. *M. caps* sMMOH, isolated from the methanotrophic bacteria *M. capsulatus*, is a homodimer of three subunits (MMOHα, MMOHβ, and MMOHγ) with the molecular weight of 215 kDa and can convert methane to methanol under the ambient condition (28). As shown in Figures. 4 and S5, the particles were well distributed on the surface of the plasma-jet-treated Neg-EM grid and comparable to those on the glow-discharge-treated grid, unlike the plasma-untreated Neg-EM grid, which showed particle/stain aggregation on the surface. This indicates that the plasma jet device can successfully introduce hydrophilicity and cleaning on the grid surface to a degree that is comparable to that introduced by a commercial glow discharge system.

### Cryo-EM analysis using the plasma-jet-treated grid

**Figure 5:**
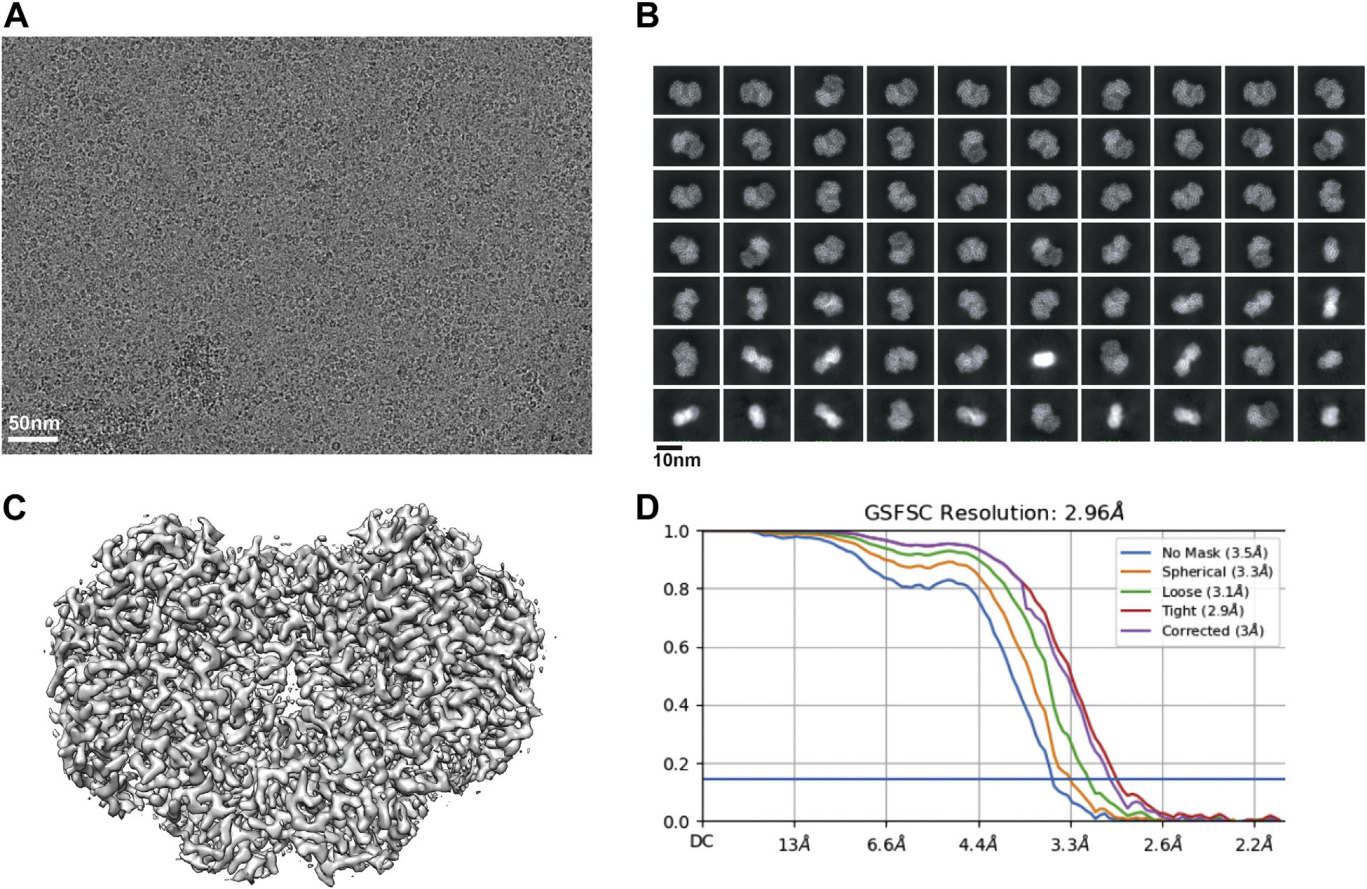
Cryo-EM analysis of *M. caps* sMMOH using the plasma-jet-treated Au Quantifoil grid. *A*, representative microscopic image of *M. caps* sMMOH. *B*, top 70 classes of the 2D classification (out of 200 classes). *C*, 3D reconstruction of *M. caps* sMMOH with a 2.96 Å resolution. Cryo-EM map was generated using the program Chimera (32, 33). *D*, the FSC_0.143_ curve adopted from cryoSPARC. cryo-EM, cryogenic electron microscopy; *M. caps* sMMOH, Methylococcus capsulatus soluble methane monooxygenase.

Plasma treatment of cryo-EM grids is a prerequisite step for specimen application and subsequent vitrification. To examine whether the plasma jet system is applicable for cryo-EM grid preparation, a gold Quantifoil grid (300 mesh, R1.2/1.3) was treated using the plasma jet (1.07 W, 60 s), and the cryo-EM grid was prepared using *M. caps* sMMOH as a test specimen. Blotting (5 s) and plunge-freezing in liquid ethane were performed using a Vitrobot mark IV (Thermo Fisher), and data collection was performed using a 300 kV Titan Krios microscope with a K3 direct electron detector. The obtained microscopic images indicate that the particles were well dispersed (Figs. 5*A* and S6). Particles showed sufficient contrast to be clearly visible under the defocus range of −1.0 to −2.0 μm, which suggests that ice thickness was well controlled in the plasma-jet-treated cryo-EM grids. A total of 2236 microscopic images were collected for particle selection and data analysis. After 2D classification (Fig. 5*B*) and the 3D reconstruction (Fig. 5*C*), we could obtain a 2.96 Å resolution structure from ∼362k particles (Fig. 5*D*). Therefore, the structural study of *M. caps* sMMOH indicated that the plasma jet is suitable for cryo-EM analysis.

In this study, we detailed an atmospheric plasma jet system that can be utilized as a surface polishing and hydrophilic treatment tool for both Neg-EM and cryo-EM grids. The plasma jet system is cost-effective, easy to set up, versatile, and most importantly, operates in an atmospheric environment without a vacuum system. We demonstrated that the plasma jet system has the potential to replace conventional glow discharge systems by comparing their effectiveness in inducing hydrophilicity, surface cleaning, and oxidation with water contact angle, AFM, and Raman spectroscopy studies.

## Experimental procedures

### Atmospheric plasma jet setup

We utilized a plasma jet, which included a dielectric barrier discharge with a guided gas flow in a tube. The necessary parts for the experimental setup are argon (Ar) gas, a DC power supply, a high voltage DC–AC inverter circuit, and a plasma jet module. The Ar gas setup and DC power supply conditions are described as follows. Compressed Ar gas (grade 4.8, purity of 99.998%) was purchased from Cryogenic Gases, and the flow was controlled by a two-stage brass regulator (CGA-580, nonflammable and nonoxidizing gas) in the range 0 to 50 psi. The gas system cost approximately $400, including a regulator ($305), cylinder stand ($67), and Ar gas ($22), with additional fees for tubing and adapters. The Ar gas flow rate was fixed at 2 psi for all plasma jet treatments. A power supply was purchased from Korad Technology (model: KD3005D) to precisely control the input power of the DC–AC converter.

The plasma jet and high voltage DC–AC inverter circuit are the key elements, which are described by Lee *et al.* (14). The power source and the plasma jet module are the same as those installed in the transdermal drug delivery device Transkin (Feagle corporation [https://feagle.co.kr/transkin]), which were purchased from the company. The specific design and experimental setup are described in Figures 1 and 2 of Lee *et al.* (14). A cylindrical metal rod with a radius of 1.5 mm was set at the center of a ceramic tube with an inner radius of 2 mm. A layer of copper tape with a width of 5 mm covered the outer ring of the alumina ceramic tube (Fig. S2). The thickness of the dielectric ceramic tube was 1 mm. The gas flow was provided through the gas inlet nozzle on the top of the plasma jet and controlled by a two-stage brass regulator. The outer electrode was grounded, and the inner electrode was connected to the high-voltage DC–AC inverter circuit, which generated a sinusoidal waveform voltage between 1 and 3 kV at 20 kHz. Because the electrode structure was asymmetric, connecting the high-voltage DC–AC inverter circuit to the opposite electrodes could produce a different result. The operation protocol was as follows. First, the gas flow was turned on and fixed to 2 psi by the regulator to maintain sufficient Ar gas flow inside the jet. Then, the DC power supply voltage was increased to generate the plasma, which was pumped out through the tube when the AC voltage was larger than the breakdown voltage between the metal rod and the ceramic tube within a ring area with an inner radius of 1.5 mm and an outer radius of 2 mm. The voltage must be maintained under a certain level to prevent the transition from a glow discharge to a streamer discharge.

### Contact angle goniometer

A contact angle goniometer (Ossila) was used to measure the water contact angle of the surface before and after plasma treatment. A water droplet was dropped on the target surface, and a polynomial curve was fit to the droplet edge with a contact angle greater than 10^°^. The tangent line at the point where the curve crossed the surface baseline was used to determine the contact angle.

### Raman spectroscopy

A Raman spectroscopy (Renishaw) system equipped with a 532 nm diode laser and a 1200 lines/mm grating was used for spectrum collection through an Olympus SLMPlan 20 × objective. All spectra were obtained in the extended scan mode in the range 3000 to 1000 cm^−1^ for analysis of framework bands, using peak positions at 2670, 1580, and 1350 cm^−1^ for analysis of the 2D, G, and D bands of graphene, respectively. Calibration of the laser was performed in static scan mode using a silicon standard.

### Atomic force microscopy (AFM)

AFM images were obtained using a Veeco Dimension Icon atomic force microscope with a ScanAsyst-Air AFM tip from Bruker Nano Inc. The data were analyzed using Nanoscope Analysis software (version 2.0).

### Negative-stain electron microscopy (Neg-EM)

Carbon-supported Neg-EM grids were prepared without (plasma untreated) and with plasma treatment. The plasma jet grid was treated with an applied power of 1 W in the ambient atmosphere, and the commercial glow discharge grid was treated with 5 mA for 1 min under vacuum (<26 mbar). *M. caps* sMMOH, purified as previously described (29), was immobilized on one of these grids, followed by the addition of uranyl formate to enhance contrast and conduct Neg-EM. Neg-EM micrographic images were collected using a 100 kV Morgagni microscope (FEI) at the University of Michigan cryo-EM center.

### Cryogenic electron microscopy (cryo-EM)

A 300 mesh Au R1.2/1.3 Quantifoil grid (Electron Microscopy Sciences) was pretreated with the plasma jet (1.07 W, 60 s). *M. caps* sMMOH (5 μl, 0.1 mg/ml) was applied to the Au Quantifoil grid and plunge-frozen using a Mark IV Vitrobot (Thermo Fisher Scientific) with 5 s of blotting time. Grids were mounted on a 300 kV Titan Krios (Thermo Fisher Scientific) using a K3 direct electron detector (with a BioQuantum energy filter, Gatan) at the Pacific Northwest Center for Cryo-EM (PNCC), and microscopic images were taken at 81,000 × with a super-resolution pixel size of 1.027 Å at the temperature of liquid nitrogen. A dose rate of 0.833 electrons/Å^2^/frame and defocus values ranging from −1.0 to −2.0 μm were used. 60 dose-fractionated movie frames resulted in an accumulated dose of 50 electrons/Å^2^. A total of 2236 movies were collected, and particle selection was performed using the Topaz program (30) embedded in the cryoSPARC software package (31). A total of 913,949 particles were selected, and 2D/3D classification was performed using the cryoSPARC software package. The final reconstructed three-dimensional structure (2.96 Å resolution) was generated from 362,535 particles (Fig. 5*C*).

## Data availability

The structural data have been deposited to the EMDB. The accession number of *M. caps* MMOH structure with the plasma-jet treated grid is EMDB-26216.

## Supporting information

This article contains supporting information.

## Conflicts of interests

The authors declare that they have no conflicts of interest with the contents of this article.
