# Supplementary material for: Development of an atmospheric plasma jet device for versatile treatment of electron microscope sample grids
Source: J Biol Chem. 2022 Mar 4;298(4):101793. doi: 10.1016/j.jbc.2022.101793 (PMC8980800; doi:10.1016/j.jbc.2022.101793)
Supplement: Supplemental Figures S1–S6 and Table S1 [file mmc1.pdf]

Supplementary Information for

Atmospheric plasma jet device for versatile electron microscope grid treatment

Eungjin Ahn, Tianyu Tang, Byungchul Kim, Hae June Lee, and Uhn-Soo Cho

Table S1. Input power conditions for plasma jet device

**Plasma jet Input conditions**	**Condition 1 (Onset power)**	**Condition 2**	**Condition 3**	**Condition 4**	**Condition 5**
Voltage (V)	7.00	7.50	10.0	12.5	15.0
Current (A)	0.078	0.085	0.107	0.123	0.14
Power (W)	0.55	0.64	1.07	1.54	2.10

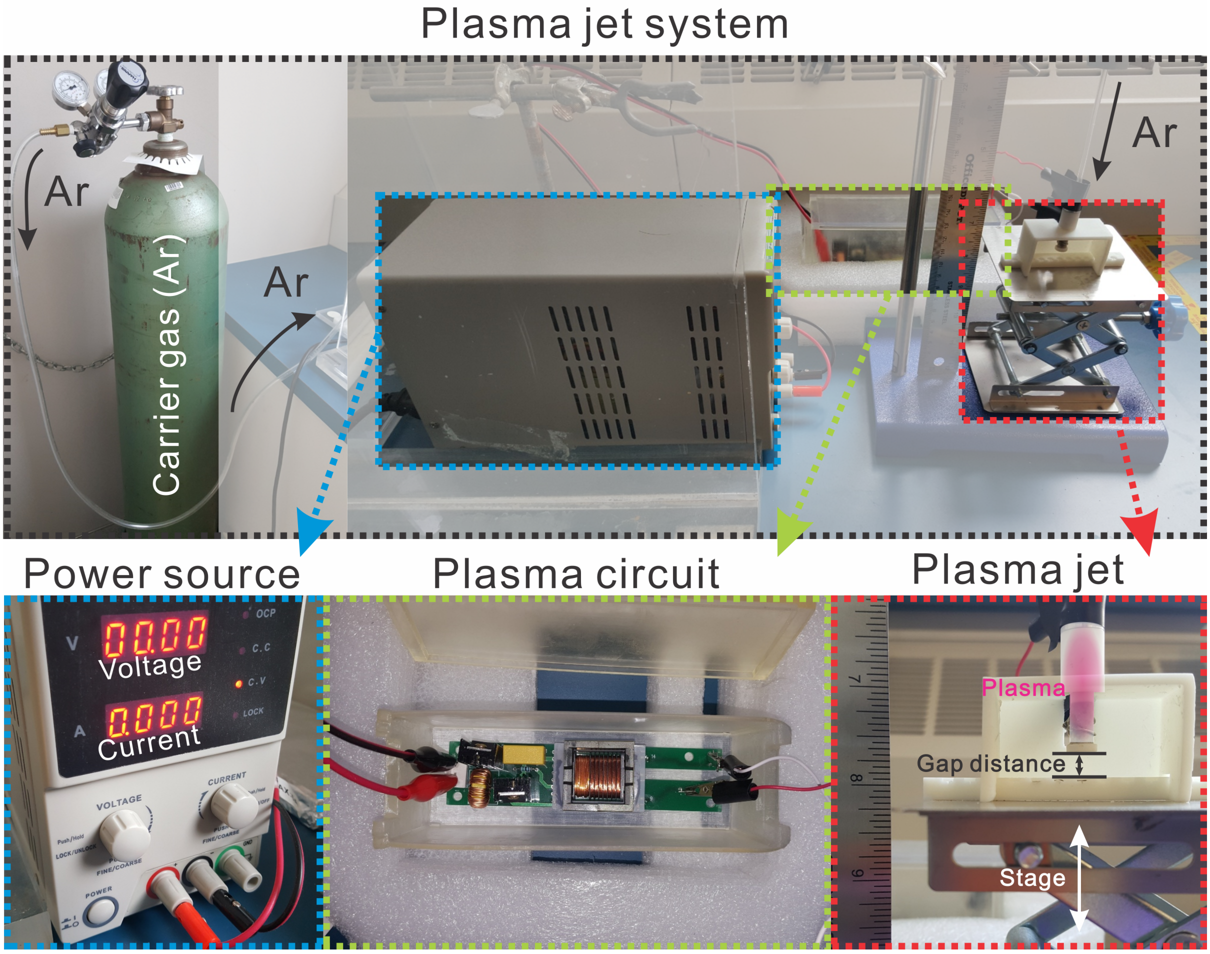

Figure S1. (a) Photograph of the overall plasma jet system (black dotted box) with each component highlighted by dotted box: power source (blue), plasma circuit (green), plasma jet (red).

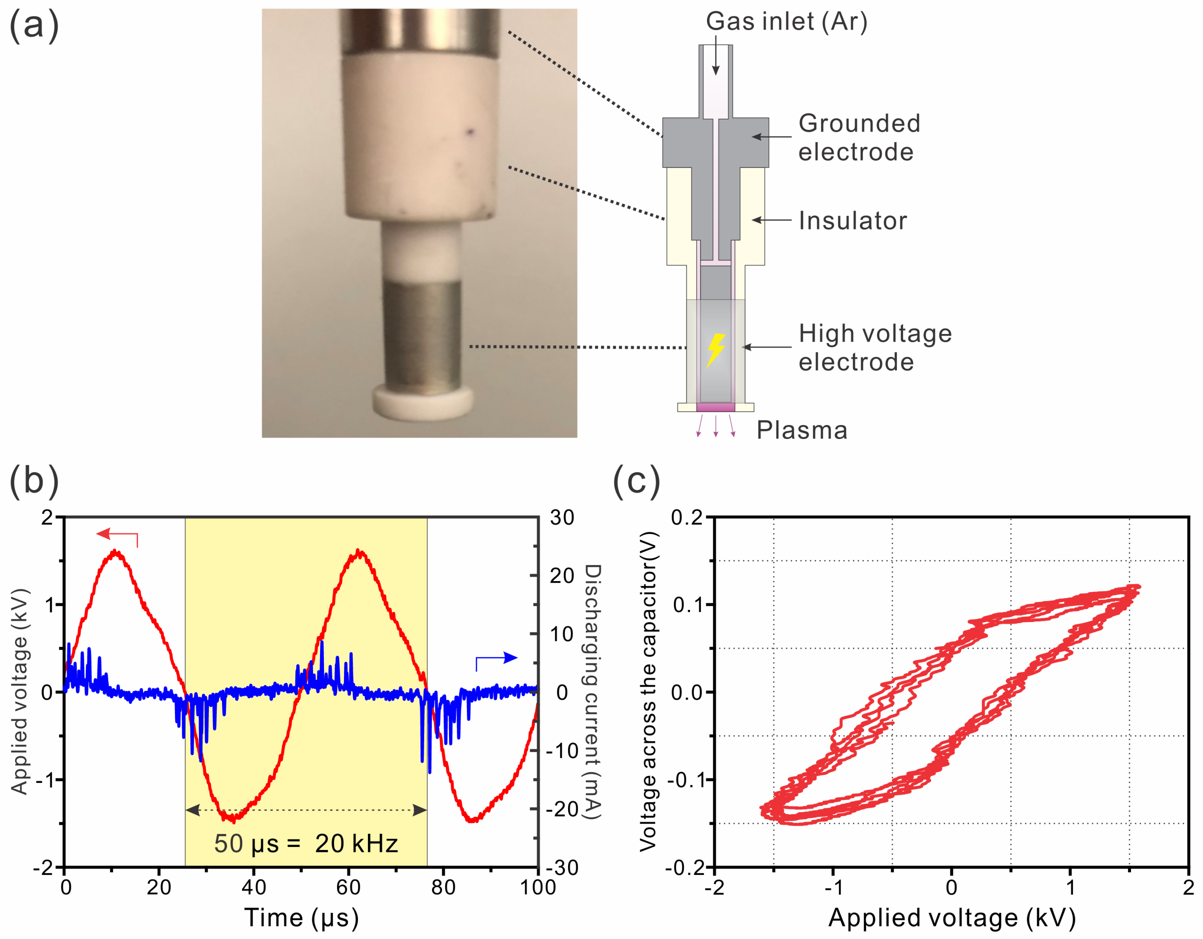

Figure S2. (a) Photograph and illustration of the inner components of the plasma jet (b) Waveforms of the applied voltage and the discharge current and (c) Lissajous figure of the plasma jet system. Applied peak-to-peak voltage and the gas flow rate are 3.2 kV and 2 SLM.

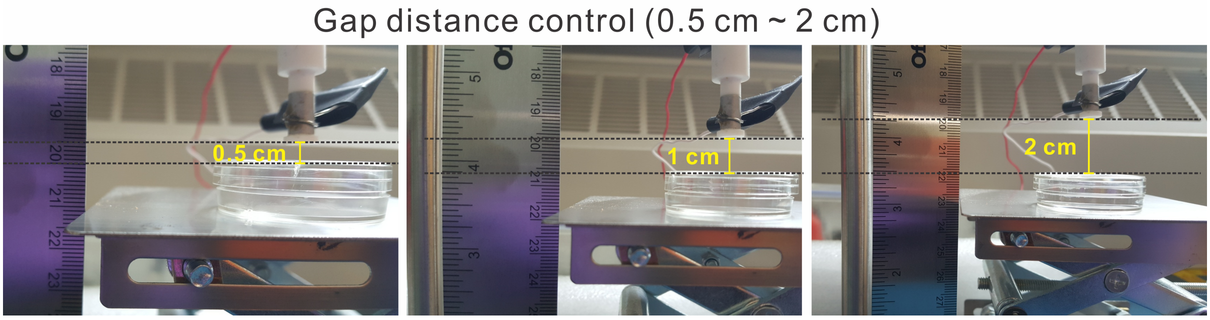

Figure S3. Photograph of plasma jet conditions on petri dish with different gap distances (0.5 cm ~ 2 cm).

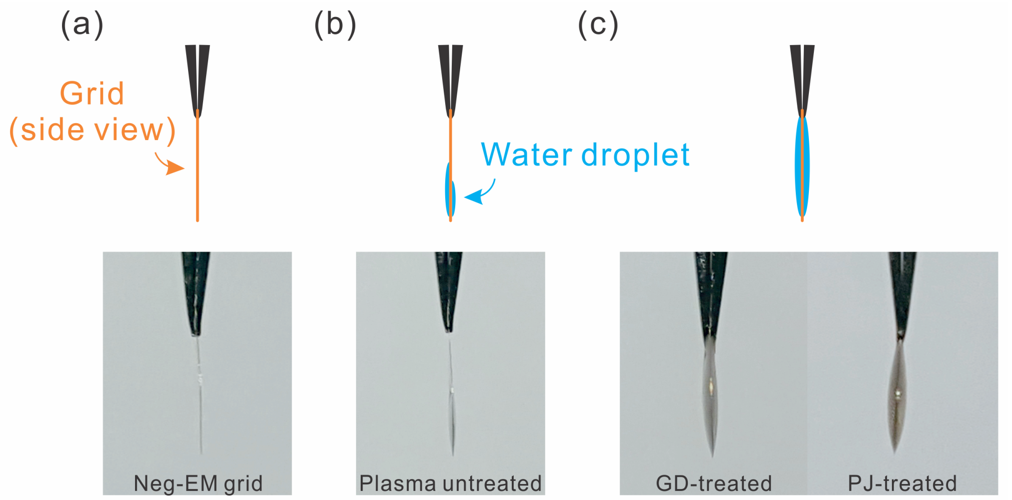

Figure S4. (a) Schematic side view and photograph of a negative-stain EM grid. Wettability test with DI water on (b) plasma untreated and (c) glow discharge (GD) and plasma jet (PJ) treated grids.

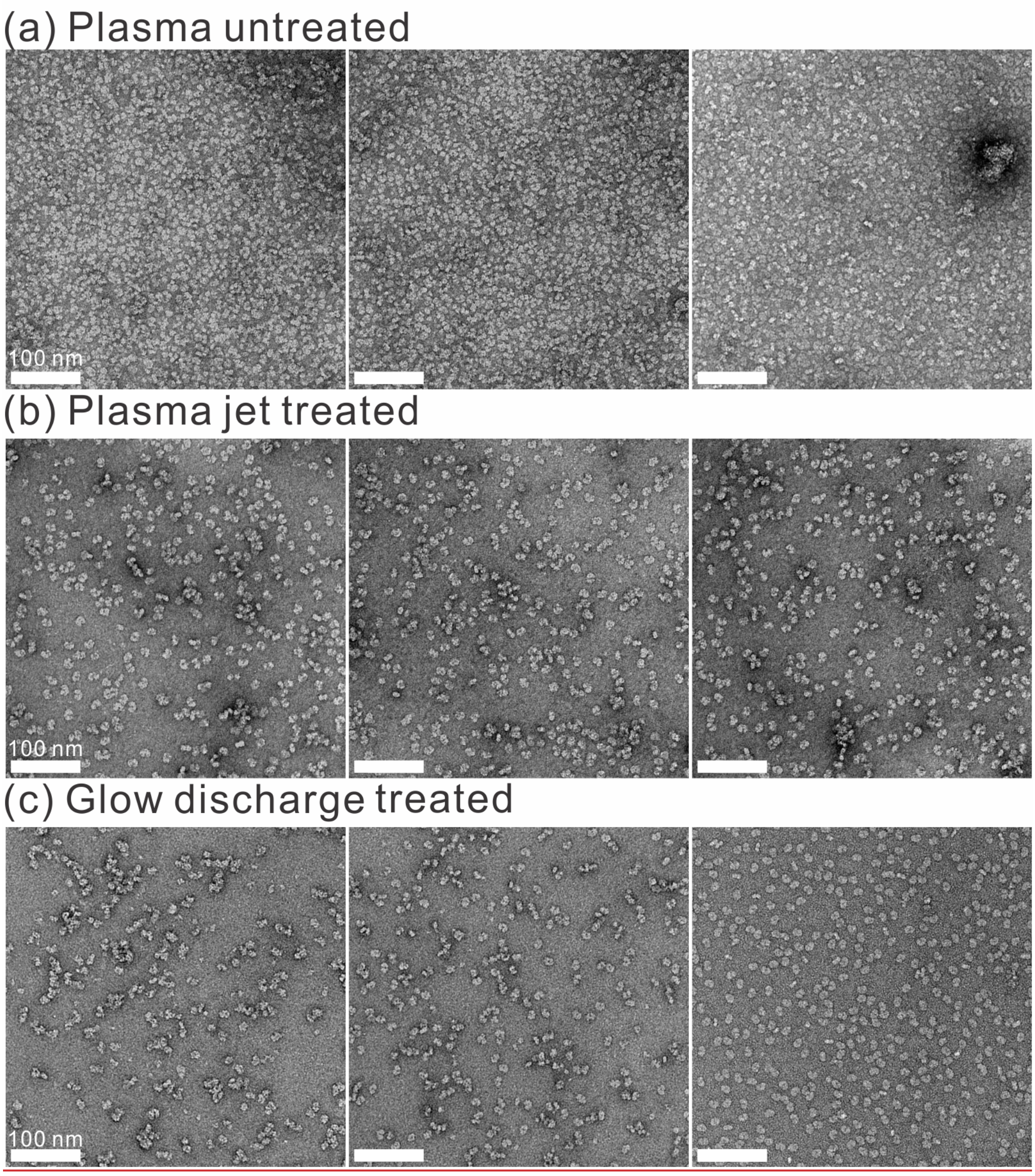

Figure S5. Negative-stain images of *M. caps* sMMOH on (a) plasma untreated, (b) plasma-jet-treated, and (c) glow-discharge-treated negative-stain grids.

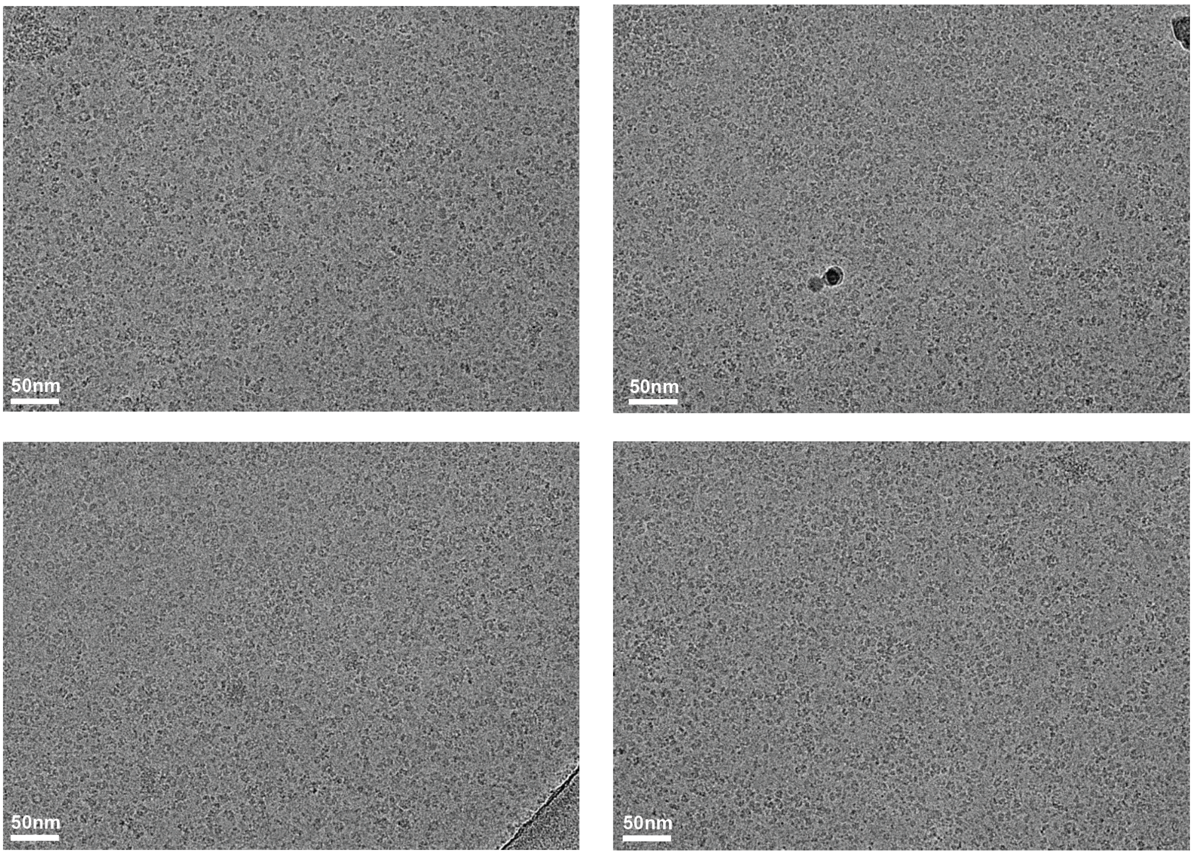

Figure S6. Additional cryo-EM microscopic images of *M. caps* sMMOH from the plasma jet-treated Au Quantifoil grid.
